# Supplementary material for: Exploring the Link Between Suicidal Concern and Pharmacotherapy in Adolescents: Evidence from a Clinical Cohort
Source: J Clin Med. 2025 Sep 11;14(18):6412. doi: 10.3390/jcm14186412 (PMC12471127; doi:10.3390/jcm14186412)
Supplement: Supplementary file 1 [file jcm-14-06412-s001.zip › jcm-3832021-supplementary.pdf]

Table S1. The RECORD statement – checklist of items, extended from the STROBE statement, that should be reported in observational studies using routinely collected health data.

	Item No.	STROBE items	Location in manuscript where items are reported	RECORD items	Location in manuscript where items are reported
**Title and abstract**					
	1	(a) Indicate the study's design with a commonly used term in the title or the abstract (b) Provide in the abstract an informative and balanced summary of what was done and what was found		RECORD 1.1: The type of data used should be specified in the title or abstract. When possible, the name of the databases used should be included.<br><br>RECORD 1.2: If applicable, the geographic region and timeframe within which the study took place should be reported in the title or abstract.<br><br>RECORD 1.3: If linkage between databases was conducted for the study, this should be clearly stated in the title or abstract.	1
**Introduction**					
Background rationale	2	Explain the scientific background and rationale for the investigation being reported			1-2
Objectives	3	State specific objectives, including any prespecified hypotheses			2
**Methods**					
Study Design	4	Present key elements of study design early in the paper			3
Setting	5	Describe the setting, locations, and relevant dates, including periods of recruitment, exposure, follow-up, and data collection			3

Participants	6	*(a) Cohort study* - Give the eligibility criteria, and the sources and methods of selection of participants. Describe methods of follow-up<br>*Case-control study* - Give the eligibility criteria, and the sources and methods of case ascertainment and control selection. Give the rationale for the choice of cases and controls<br>*Cross-sectional study* - Give the eligibility criteria, and the sources and methods of selection of participants<br><br>*(b) Cohort study* - For matched studies, give matching criteria and number of exposed and unexposed<br>*Case-control study* - For matched studies, give matching criteria and the number of controls per case		RECORD 6.1: The methods of study population selection (such as codes or algorithms used to identify subjects) should be listed in detail. If this is not possible, an explanation should be provided.<br><br>RECORD 6.2: Any validation studies of the codes or algorithms used to select the population should be referenced. If validation was conducted for this study and not published elsewhere, detailed methods and results should be provided.<br><br>RECORD 6.3: If the study involved linkage of databases, consider use of a flow diagram or other graphical display to demonstrate the data linkage process, including the number of individuals with linked data at each stage.	3-4
Variables	7	Clearly define all outcomes, exposures, predictors, potential confounders, and effect modifiers. Give diagnostic criteria, if applicable.		RECORD 7.1: A complete list of codes and algorithms used to classify exposures, outcomes, confounders, and effect modifiers should be provided. If these cannot be reported, an explanation should be provided.	4-5
Data sources/ measurement	8	For each variable of interest, give sources of data and details of methods of assessment (measurement).<br>Describe comparability of assessment methods if there is more than one group			4-5

Bias	9	Describe any efforts to address potential sources of bias			3
Study size	10	Explain how the study size was arrived at			3-4
Quantitative variables	11	Explain how quantitative variables were handled in the analyses. If applicable, describe which groupings were chosen, and why			4-5
Statistical methods	12	(a) Describe all statistical methods, including those used to control for confounding<br>(b) Describe any methods used to examine subgroups and interactions<br>(c) Explain how missing data were addressed<br>(d) *Cohort study* - If applicable, explain how loss to follow-up was addressed<br>*Case-control study* - If applicable, explain how matching of cases and controls was addressed<br>*Cross-sectional study* - If applicable, describe analytical methods taking account of sampling strategy<br>(e) Describe any sensitivity analyses			7
Data access and cleaning methods		..		RECORD 12.1: Authors should describe the extent to which the investigators had access to the database population used to create the study population.	3, 7

				RECORD 12.2: Authors should provide information on the data cleaning methods used in the study.	
Linkage		..		RECORD 12.3: State whether the study included person-level, institutional-level, or other data linkage across two or more databases. The methods of linkage and methods of linkage quality evaluation should be provided.	
**Results**					
Participants	13	(a) Report the numbers of individuals at each stage of the study (*e.g.*, numbers potentially eligible, examined for eligibility, confirmed eligible, included in the study, completing follow-up, and analysed)<br>(b) Give reasons for non-participation at each stage.<br>(c) Consider use of a flow diagram		RECORD 13.1: Describe in detail the selection of the persons included in the study (*i.e.,* study population selection) including filtering based on data quality, data availability and linkage. The selection of included persons can be described in the text and/or by means of the study flow diagram.	5-7
Descriptive data	14	(a) Give characteristics of study participants (*e.g.*, demographic, clinical, social) and information on exposures and potential confounders<br>(b) Indicate the number of participants with missing data for each variable of interest<br>(c) *Cohort study* - summarise follow-up time (*e.g.*, average and total amount)			5-7
Outcome data	15	*Cohort study* - Report numbers of outcome events or summary measures over time<br>*Case-control study* - Report numbers in each exposure			7-9

		category, or summary measures of exposure<br>*Cross-sectional study* - Report numbers of outcome events or summary measures			
Main results	16	(a) Give unadjusted estimates and, if applicable, confounder-adjusted estimates and their precision (e.g., 95% confidence interval). Make clear which confounders were adjusted for and why they were included<br>(b) Report category boundaries when continuous variables were categorized<br>(c) If relevant, consider translating estimates of relative risk into absolute risk for a meaningful time period			7-9
Other analyses	17	Report other analyses done—e.g., analyses of subgroups and interactions, and sensitivity analyses			9-10
**Discussion**					
Key results	18	Summarise key results with reference to study objectives			10-11
Limitations	19	Discuss limitations of the study, taking into account sources of potential bias or imprecision. Discuss both direction and magnitude of any potential bias		RECORD 19.1: Discuss the implications of using data that were not created or collected to answer the specific research question(s). Include discussion of misclassification bias, unmeasured confounding, missing data, and changing eligibility over time, as they pertain to the study being reported.	11-12
Interpretation	20	Give a cautious overall interpretation of results considering objectives,			10-12

		limitations, multiplicity of analyses, results from similar studies, and other relevant evidence			
Generalisability	21	Discuss the generalisability (external validity) of the study results			10-12
**Other Information**					
Funding	22	Give the source of funding and the role of the funders for the present study and, if applicable, for the original study on which the present article is based			12
Accessibility of protocol, raw data, and programming code		..		RECORD 22.1: Authors should provide information on how to access any supplemental information such as the study protocol, raw data, or programming code.	12

*Reference: Benchimol EI, Smeeth L, Guttmann A, Harron K, Moher D, Petersen I, Sørensen HT, von Elm E, Langan SM, the RECORD Working Committee. The REporting of studies Conducted using Observational Routinely-collected health Data (RECORD) Statement. *PLoS Med* 2015; 12(10): e1001885. doi:10.1371/journal.pmed.1001885 .[61]

*Checklist is protected under Creative Commons Attribution (CC BY) license.

Table S2. Sociodemographic, diagnostic, and pharmacological data of the low and high-risk groups

Variable	Low Risk	High Risk	*p*	Effect Size
Females	26 (86.7%)	102 (87.9%)	0.765	0.000
Males	4 (13.3%)	14 (12.1%)	0.765	0.000
Age M±SD	15.62 ± 1.3	15.46 ± 1.58	0.580	0.102
Ethnicity			0.884	0.131
Caucasian	28 (93.3%)	100 (87.7%)		
Asian	0 (0%)	3 (2.6%)		
African	1 (3.3%)	4 (3.5%)		
Latina	0 (0%)	4 (3.5%)		
Mixed	1 (3.3%)	3 (2.6%)		
Social relations			0.158	0.156
Social withdrawal	1 (3.4%)	16 (14%)		
Poor	14 (48.3%)	59 (52.7%)		
Adequate	14 (48.3%)	39 (34.2%)		
School performance			0.376	0.171
Poor	6 (20.7%)	15 (13%)		
Sufficient	6 (20.7%)	32 (27.8%)		
Good	7 (24.1%)	43 (37.4%)		
Excellent	5 (17.2%)	14 (12.2%)		
Withdrawal	5 (17.2%)	11 (9.6%)		
Risky behaviors			0.002**	0.378
No	12 (42.9%)	18 (15.8%)		
Non-suicidal self-injury (e.g., cutting)	13 (46.4%)	50 (43.9%)		
Drug use	1 (3.6%)	4 (3.5%)		
Suicidal behaviors	1 (3.6%)	20 (17.5%)		
Non-suicidal self-injury + drugs	1 (3.6%)	0 (0%)		
Non-suicidal self-injury + suicidal behaviors	0 (0%)	19 (16.7%)		
Non-suicidal self-injury + drugs + suicidal behaviors	0 (0%)	3 (2.6%)		
Diagnoses				
Neurodevelopmental disorders [a]	2 (6.7%)	13 (11.2%)	0.736	0.033
Psychotic disorders/symptoms [b]	5 (16.7%)	32 (27.6%)	0.322	0.082
Bipolar disorders - present	1 (3.3%)	4 (3.4%)	1.000	0.000
Depressive disorders [c]	18 (60%)	67 (58.3%)	1.000	0.000
Anxiety disorders [d]	12 (40%)	37 (32.2%)	0.555	0.049
OCD	2 (6.7%)	4 (3.5%)	0.604	0.022
PTSD	2 (6.7%)	5 (4.3%)	0.634	0.004
Somatic disorder	0 (0%)	3 (2.6%)	1.000	0.014
Eating disorders [e]	14 (46.7%)	24 (20.9%)	0.007**	0.218
Sleep disorders [f]	1 (3.3%)	1 (0.9%)	0.372	0.013
Gender dysphoria	0 (0%)	2 (1.7%)	1.000	0.000
Conduct disorders [g]	3 (10%)	2 (1.7%)	0.060	0.137
Substance use disorder	1 (3.3%)	1 (0.9%)	0.372	0.013
Personality disorders [h]	6 (20%)	35 (30.4%)	0.367	0.075
Other [i]	4 (13.3%)	32 (27.8%)	0.153	0.116
Medications at T0	25 (83.3%)	83 (71.6%)	0.281	0.089
Antipsychotics at T0	10 (34.5%)	44 (40.4%)	0.717	0.031
Antidepressants at T0	9 (31%)	42 (38.5%)	0.624	0.073
Benzodiazepines at T0	11 (37.9%)	45 (41.3%)	0.909	0.010

Mood stabilizers at T0	2 (6.9%)	9 (8.3%)	1.000	0.000
Supplements [j] at T0	15 (51.7%)	37 (33.6%)	0.115	0.134
Medication duration before T0	13.05 ± 19.64	10.89 ± 16.14	0.646	0.128
Medications at T1	26 (86.7%)	101 (89.4%)	0.745	0.008
Antipsychotics at T1	14 (46.7%)	57 (52.3%)	0.734	0.029
Antidepressants at T1	12 (40%)	55 (49.5%)	0.470	0.061
Benzodiazepines at T1	11 (36.7%)	53 (47.7%)	0.382	0.074
Mood stabilizers at T1	1 (3.3%)	13 (11.7%)	0.301	0.086
Supplements at T1	13 (43.3%)	55 (49.5%)	0.690	0.034
CGI-S	4.85 ± 1.19	4.71 ± 0.93	0.592	0.138
CGAS	50.04 ± 9.33	48.44 ± 11.83	0.471	0.141

Significance: ** $p < 0.01$. Note: [a] ADHD, Learning disabilities, Tic; [b] Psychosis, Attenuated Psychotic Syndrome, Unspecified Schizophrenia Spectrum and Other Psychotic Disorder, Clinical High Risk for Psychosis; [c] Major Depressive Disorder, Dysthymia, Disruptive Mood Dysregulation Disorder; [d] Generalized Anxiety Disorder, Separation Anxiety; [e] Anorexia Nervosa, Bulimia Nervosa, Binge-eating Disorder, Unspecified Eating Disorders; [f] Insomnia, Circadian Rhythm Disorder; [g] Oppositive Defiant Disorder, Conduct Disorder; [h] Structuring personality disorders, since diagnosing a personality disorders in children and adolescents is not always appropriate, minors who exhibit characteristics of subthreshold personality disorders but do not fully meet the criteria of the DSM-5 are diagnosed with structuring personality disorders; [i] Headaches, Migraines; [j] Dietary supplements, melatonin, passion flowers.

Table S3. Differences between psychotropic prescriptions at T1 - depression group (n = 100)

Medication	NSC		SC		
	n	%	*n*	%	*p*
Antipsychotics at T1	4	26.7	39	47.6	0.165
Antidepressants at T1	8	53.3	50	59.5	0.778
Benzodiazepines at T1	5	33.3	39	46.4	0.408
Mood stabilizers at T1	0	0.0	9	10.7	0.348
Supplements at T1	5	33.3	40	47.6	0.402
Total medications prescribed	12	80.0	77	90.6	0.363

Table S4. Differences between psychotropic prescriptions at T1 – depression/anxiety group (n = 117)

Medication	NSC		SC		
	n	%	*n*	%	*p*
Antipsychotics at T1	4	19.0	45	49.5	0.014*
Antidepressants at T1	13	61.9	52	55.9	0.808
Benzodiazepines at T1	7	33.3	43	46.2	0.336
Mood stabilizers at T1	0	0.0	9	9.7	0.207
Supplements at T1	6	28.6	45	48.4	0.144
Total medications prescribed	17	73.9	86	91.5	0.031*

Significance: * $p < 0.05$

Table S5. Changes in prescriptions at T0 and T1 – depression group

Medication	Group	T0 to T1	T1 to T0	χ^2	*p*
Antidepressants	No concern	4	0	2.250	0.134
	Concern	16	6	3.682	0.055
Antipsychotics	No concern	2	1	0.000	1.000
	Concern	11	4	2.400	0.121
Benzodiazepines	No concern	0	2	0.500	0.480
	Concern	11	6	0.941	0.332
Mood Stabilizers	No concern	0	0	-	-
	Concern	3	0	1.333	0.248
Supplements	No concern	4	2	0.167	0.683
	Concern	18	8	3.115	0.078
Total medications	No concern	2	0	0.500	0.480
prescribed	Concern	16	2	9.389	0.002**

Significance: ** $p < 0.01$; ***

Table S6. Changes in prescriptions at T0 and T1 – depression/anxiety group

Medication	Group	T0 to T1	T1 to T0	χ^2	*p*
Antidepressants	No concern	8	0	6.125	0.013*
	Concern	17	6	4.348	0.037*
Antipsychotics	No concern	2	2	0.000	1.000
	Concern	13	4	3.765	0.052
Benzodiazepines	No concern	1	2	0.000	1.000
	Concern	13	7	1.250	0.264
Mood Stabilizers	No concern	0	0	-	-
	Concern	3	1	0.250	0.617
Supplements	No concern	4	3	0.000	1.000
	Concern	21	8	4.966	0.026*
Total medications prescribed	No concern	4	1	0.800	0.371
	Concern	20	2	13.136	<0.001***

Significance: * $p < 0.05$; *** $p < 0.001$

Table S7. Logistic linear regressions between pharmacological prescriptions and groups – depression and depression/anxiety

Group	Medication	OR	*p*
Depression	Antipsychotics at T1	1.658	0.463
	Antidepressants at T1	1.723	0.495
	Benzodiazepines at T1	1.286	0.731
	Supplements at T1	1.694	0.446
	Total medications prescribed at T1	2.504	0.286
Depression/anxiety	Antipsychotics at T1	0.880	0.838
	Antidepressants at T1	5.246	0.033*
	Benzodiazepines at T1	1.202	0.778
	Supplements at T1	1.647	0.412
	Total medications prescribed at T1	4.354	0.052

Significance: * $p < 0.05$
